# Effectiveness of a Short-Term Treatment of Oxygen-Ozone Therapy into Healing in a Posttraumatic Wound

**DOI:** 10.1155/2016/9528572

**Published:** 2016-08-22

**Authors:** Irene Degli Agosti, Elena Ginelli, Bruno Mazzacane, Gabriella Peroni, Sandra Bianco, Fabio Guerriero, Giovanni Ricevuti, Simone Perna, Mariangela Rondanelli

**Affiliations:** ^1^Rehabilitation Unit, Azienda di Servizi alla Persona di Pavia, 27100 Pavia, Italy; ^2^Department of Public Health, Experimental and Forensic Medicine, School of Medicine, Endocrinology and Nutrition Unit, University of Pavia, Pavia, Italy; ^3^Section of Geriatrics, Department of Internal Medicine and Medical Therapy, University of Pavia, 27100 Pavia, Italy

## Abstract

*Introduction*. A number of studies suggest that oxygen-ozone therapy may have a role in the treatment of chronic, nonhealing, or ischemic wounds for its disinfectant and antibacterial properties. Nonhealing wounds are a significant cause of morbidity. Here we present a case of subcutaneous oxygen-ozone therapy used to treat a nonhealing postoperative wound in a young man during a period of 5 weeks.* Case Presentation*. A 46-year-old man had a motorcycle accident and underwent amputation of the right tibia and fibula. At the discharge he came to our attention to start rehabilitation treatment. At that time the wound was ulcerated but it was afebrile with no signs of inflammation and negativity to blood tests. At 2 months from the trauma despite appropriate treatment and dressing, the wound was slowly improving and the patient complained of pain. For this reason in addition to standard dressing he underwent oxygen-ozone therapy. After 5 weeks of treatment the wound had healed.* Conclusion*. In patients with nonhealing wounds, oxygen-ozone therapy could be helpful in speeding the healing and reducing the pain thanks to its disinfectant property and by the increase of endogenous oxygen free radicals' scavenging properties. Compared to standard dressing and other treatments reported in the literature it showed a shorter time of action.

## 1. Introduction

The treatment of wounds and major traumatic amputations is a clinical challenge due to high treatment costs, high infection rates, slow healing, and resulting handicaps. Traumatic amputations are almost always contaminated with any sort of pathogen and the infection rates are associated with the severity of soft tissue damage. Moreover, the reperfusion syndrome impairs the restored microcirculatory perfusion and local delivery of antibiotics due to endothelial leakage and precapillary shunting [1–3]. Chronicity of a wound is due to an imbalance between local tissue demand and systemic metabolic supply resulting in tissue inflammation, anoxia, oedema, induration, and extravasation of cytokines and blood formed elements. The final step of this pathophysiological cascade is cellular death. This condition is characterized by a lack of physiological antioxidant defence mechanisms and an increase in free radical production [3]. The wound healing in any tissue follows several stages that are the inflammatory phase, the migratory phase, the proliferative phase, and the remodelling phase [2–5]. Currently there are several effective approaches to treat wounds, such as topical antimicrobial agents, surgical and enzymatic debriding agents, collagen or alginate dressings, intermittent pneumatic compression, topically applied mesoglycan, keratinocyte growth factor 2, and topical negative pressure. However, an effective method able to promote healing and prevent relapse is not available [1, 6–12].

Today oxygen-ozone therapy is recognised to have a disinfectant property and to induce a strong oxidative stress which stimulates the protective mechanisms of cells and organs increasing the efficacy of endogenous oxygen free radicals' scavenging properties [3, 13–19]. The antibacterial properties of oxygen-ozone therapy have been studied in detail and have been extensively reported in dental and other literature [16, 17, 20, 21].

Oxygen-ozone therapy inactivates bacteria disrupting their cell envelope through oxidation of the phospholipids and lipoproteins, inhibits fungi growth, damages the capsid of viruses, and upsets the reproductive cycle by disrupting the virus-to-cell contact with peroxidation. Oxygen-ozone therapy causes an increase in the red blood cell glycolysis rate, causing the stimulation of 2,3-diphosphoglycerate which leads to an increase of oxygen released to the tissues and activates the Krebs cycle stimulating production of ATP. It also causes a reduction in NADH and helps to oxidize cytochrome C. There is a stimulation of production of prostacyclin, a vasodilator, and of enzymes which act as free radical scavengers and cell-wall protectors: glutathione peroxidase, catalase, and superoxide dismutase. Then it increases the production of interferon, tumor necrosis factor, and interleukin-2, activating the immune system [17, 19]. Today oxygen-ozone therapy is recognised to have a role along with standard treatments as is highlighted by other studies [1, 3].

Here we present a case of a young man after surgical treatment of posttraumatic accident. His ulcer was poorly reacting to standard dressing for two months. Adjuvant combined oxygen-ozone therapy was used in this patient with good results. The aim of the study was to observe the effect of subcutaneous oxygen-ozone injections, by the healing of postsurgical wound. The patient was subjected to subcutaneous oxygen-ozone injections after undressing and skin disinfection. At every week a picture of the wound was taken to measure the size. Each treatment session was repeated daily until the wound healed.

## 2. Case Report

The patient, P.G., a 46-year-old man, married, had a motorcycle accident and underwent amputation of the right tibia and fibula. He reported a history of smoking, high blood pressure, allergy to amoxicillin, and previous appendectomy. 21 days after the surgery in response to the TAC of fluid collection in the residual limb he underwent surgical revision of the stump.

The control of wound was subjected to anesthesiologic visit during the follow-up. At discharge it was afebrile with no signs of inflammation to the blood tests. The suggested therapy was Gabapentin 300 mg qid, Oxycodone 15 mg bid, Clonazepam 5 gg/die, Omeprazole 20 mg/die, Seleparina 0,4 mg 1 fl/die, ferrous sulphate 1 cp/die, vitamin C, and folic acid 1 cp/die; when the pain was uncontrolled, the suggested therapy was 1 to 3 cp/die or Oxycodone + Acetaminophen 5 mg 1 to 3 cp/die. Three days later he came to our institute to start the rehabilitation treatment. Despite appropriate treatment with drugs and dressing, the wound was slowly improving and the patient complained of pain. After 2 months the wound was not yet healed and oxygen-ozone therapy was proposed and started after signing informed consent and ethics committee approval. Meanwhile he continued rehabilitation sessions and underwent standard dressing until the wound had healed.

### 2.1. Oxygen-Ozone Therapy Intervention

The treatment consisted of subcutaneous medical oxygen-ozone injection around the wound before undressing and skin disinfection. The treatment lasted for 5 weeks (Table 1).

Medical oxygen-ozone, an ozone/oxygen mixture consisting of purest oxygen-ozone therapy was produced on-site from medical oxygen (in accordance with pharmaceutical legislation) using a medical ozone generator [22–24].

#### 2.1.1. First Stage/Week

**Figure 1:**
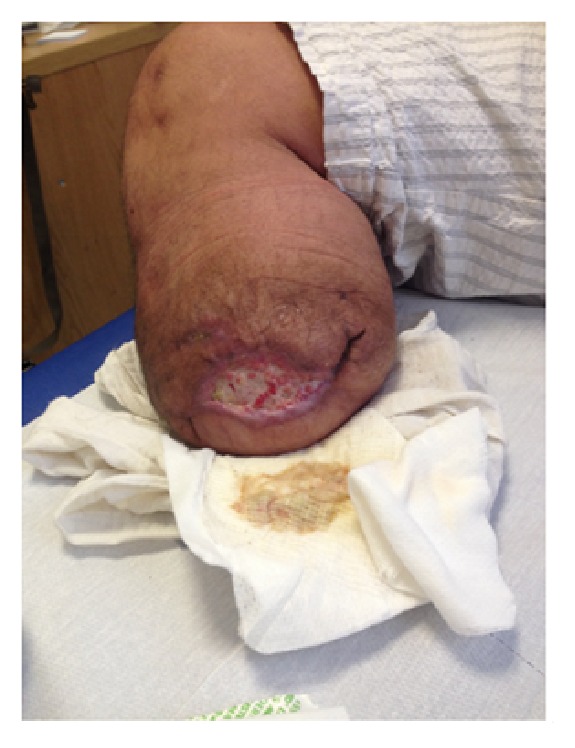
Stage I at 1st week.

At the beginning the patient was afebrile with no signs of inflammation and negativity to blood tests but he complained of pain for which he took Gabapentin 300 mg qid and Oxycodone 15 mg bid. The wound was ulcerated with a size of 6,5 cm (Figure 1).

During the first week the patient was treated with 8 *μ*g of medical oxygen-ozone. At the end of the first week of treatment sessions the wound was 4 cm of size and the patient halved the dose of analgesics and from the second session he reported a reduction of phantom limb pain.

#### 2.1.2. Second Week

**Figure 2:**
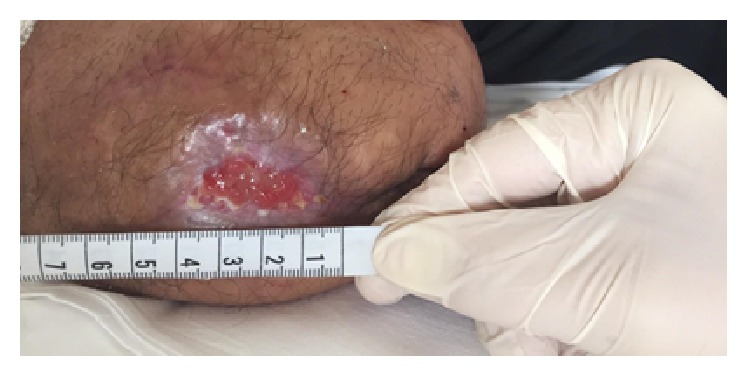
Stage II at 2nd week.

During the second week he was treated with 18 *μ*g of oxygen-ozone. At the 10th session the wound measured 3,5 cm and the patient reported a feeling of wellness and temporally stopped the oral drugs (Figure 2).

#### 2.1.3. Third Week

**Figure 3:**
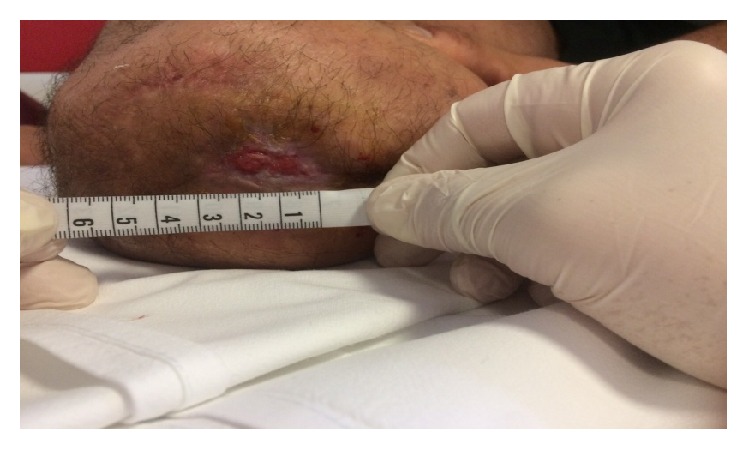
Stage III at 3rd week.

**Figure 4:**
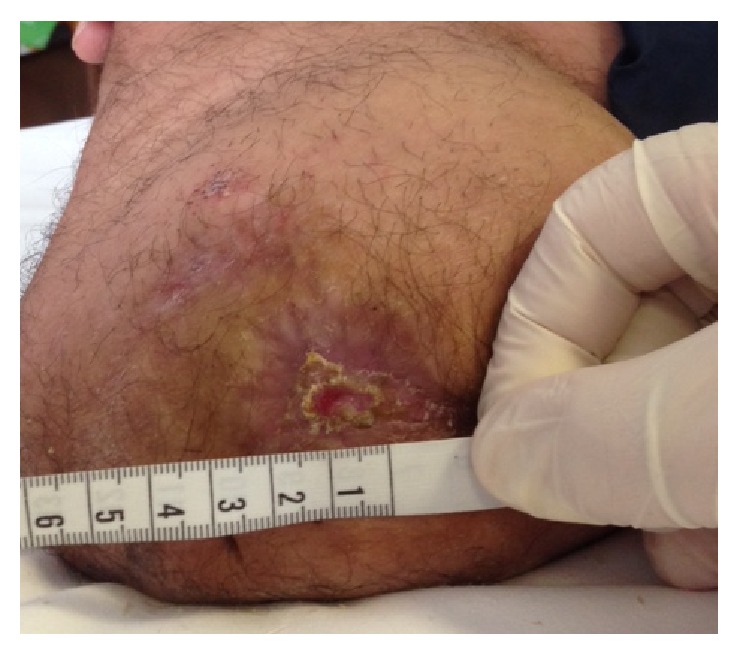
Stage IV at 4th week.

In the third week of treatment we injected the wound with 24 *μ*g of medical oxygen-ozone. At the beginning of this period the wound measured 2,5 cm and at the end of the week it reduced to 1,9 cm (Figures 3 and 4). Meanwhile the patient gradually reduced the intake of analgesics up to taking them only when needed. He was referred for taking Gabapentin 600 mg/die and Oxycodone 5 mg/die.

#### 2.1.4. Fourth Week

**Figure 5:**
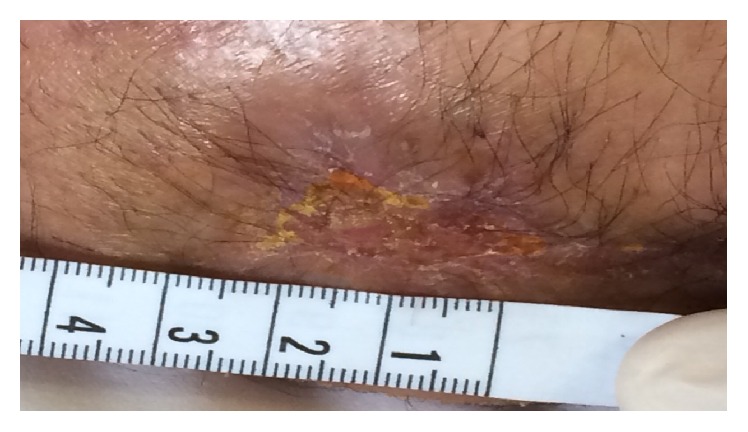
Stage V at 5th week.

At the fourth week he was treated with 14 *μ*g of oxygen-ozone. At the end of this period the ulcer was 1 cm and the patient reported continuing taking the oral analgesics only when needed (Figure 5). He did not report phantom limb pain or nausea as a side effect of opioids.

#### 2.1.5. Fifth Week

**Figure 6:**
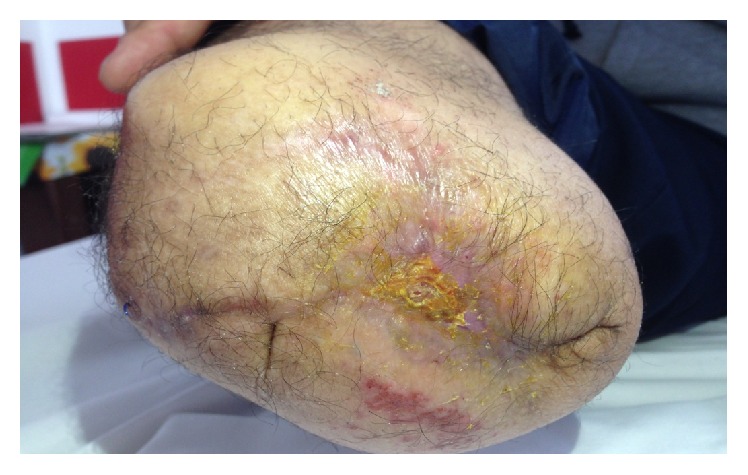
Stage VI at 6th week.

During the last days of treatment he was cured with 6 *μ*g of oxygen-ozone. At the fifth session the wound had healed (Figure 6).

**Figure 7:**
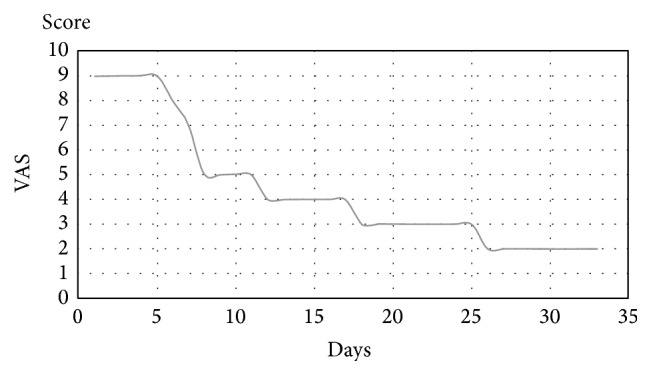
VAS score during treatment.

During the treatment the patient was asked daily to report his pain with the VAS (Visual Analogic Scale). The pain remained constant during the first week and gradually reduced during the following period (Figure 7).

## 3. Discussion

Every year millions of people worldwide are affected by poor wound healing after trauma, surgery, acute illness, or chronic disease conditions. This is the consequence of poorly regulated elements of the healthy tissue repair response, including inflammation, angiogenesis, matrix deposition, and cell recruitment. Experimental evidences suggest that the healing process in the chronic wounds is obstructed by local ischaemia due to hypoxia, lactic acid, reactive oxygen species, and proinflammatory cytokines.

Medical O_3_ is used in different ways to disinfect and to treat diseases, infections, and wounds since 150 years [17].

For example, the autologous infusion of ozonated blood is able to restore physiological pH and the production of critical growth factors; moreover, the Nrf2 activation promotes the production of phase II proteins, antioxidant proteins, and an enhanced release of GSH, thioredoxin, and NADPH. Consequently the normalization of the antioxidant-redox cycling and the detoxification system slowly favours the healing and tissue regeneration [1, 25, 26]. It has been noticed that oxygen-ozone therapy increases the collagen contents of the wounds and upregulates levels of VEGF, TGF-*β*, and PDGF in wound exudates [27, 28]. Some studies have showed that the treatment with oxygen-ozone is an adjuvant to the conventional modality for treatment of extensive orthopaedic wounds [29, 30].

According to this literature our case showed the possible effectiveness of a short-term treatment with oxygen-ozone into healing in a posttraumatic wound. In less than 5 weeks, that is, 33 days, we noticed a complete resolution of the tear combining subcutaneous injections of oxygen-ozone and standard dressing. Other studies reported the efficacy of oxygen-ozone by using different methods, that is, oxygen-ozone bags and AHT (autohemotransfusion) at higher dosage and superficial intermittent oxygen-ozone application [29, 30].

Currently as shown by other studies the average healing time of complex wound is 45 days with the classic dressings 12, from three to eight weeks with hyaluronic acid [31].

In literature, in patients treated with platelet rich plasma (PRP) and hyaluronic acid dressing and with hyaluronate-iodine complex, the mean healing time was 18 weeks [32, 33].

We have seen fast healing probably due to upregulation of genes responsible for the transcription of antioxidant proteins, phase II enzymes and heme-oxygenase-1, a release of oxygen, and growth factor [27, 28, 34].

During the treatment the patient reported a feeling of wellness, which is likely due to a stimulation of the neuroendocrine system with a transitory increase of adrenocorticotrophic hormone-cortisol, serotonin, and endorphins [20].

He also referred to a reduction of the nausea for halving the opioids dosage. The strength of our study can be related to the young age of the patient and the absence of inflammation and negativity to blood tests when he came to our attention. A limitation can be found in the absence of similar cases to compare the results. In the literature, there are no specific guidelines for the treatment of postsurgical wound with subcutaneous oxygen-ozone injection. Although oxygen-ozone is widely used, it is well known as a toxic gas in the troposphere and there is a lack of reliable scientific reports [21].

Recent advances in the molecular and cellular aspects of redox biology positions as well revisit the apparently outstanding benefit of oxygen therapy in wound healing. It is likely that reactive derivatives of molecular oxygen, oxidants, for example, serve as cellular messengers to support the healing process. Strategies to manipulate the oxygen/oxidant environment in the wound are likely to be of outstanding significance [35].

Wound healing is a complex process; therefore, it is not possible to determine exactly what percentage of oxygen-ozone is responsible for it but, according to the literature, our case report confirms a possible positive involvement of oxygen-ozone in promoting the wound healing when traditional treatments alone are not adequate. In conclusion, we can say that, in agreement with the literature, this report suggests a positive role of oxygen-ozone therapy in promoting wound healing and controlling pain of complicated wounds. Anyway other studies are needed to confirm the effectiveness as an adjuvant to the conventional modality for treatment.

## Figures and Tables

**Table 1:** Progression, dosage, and clinical aspects of the treatment.

Progression	Dosage (average)	Wound size	Clinical aspects
1st week	7 *μ*g	6,5 cm	The patient took Gabapentin 300 mg qid and Oxycodone 15 mg bid; from the second session he reported a reduction of phantom limb pain

2nd week	18 *μ*g	3,5 cm	The patient temporally stopped oral therapy; he referred to a feeling of wellness

3rd week	24 *μ*g	2,5 cm (at the 1st session of the week), 1,9 cm (at the end of the week)	Good control of the pain; oral therapy only when needed (600 mg Gabapentin/die + 5 mg Oxycodone/die)

4th week	14 *μ*g	1 cm	No more phantom limb pain, no nausea

5th week	6 *μ*g	Closed (at the 5th day of the week)	Good control of the pain, feeling of wellness
